# Predictive value of the maximum serum creatinine value and growth rate in acute paraquat poisoning patients

**DOI:** 10.1038/s41598-018-29800-0

**Published:** 2018-08-02

**Authors:** Meng-Xiao Feng, Yu-Ning Li, Wei-Shuyi Ruan, Yuan-Qiang Lu

**Affiliations:** 1 Department of Emergency Medicine, the First Affiliated Hospital, School of Medicine, Zhejiang University, Hangzhou, People’s Republic of China; 2 School of Mathematical Sciences, Zhejiang University, Hangzhou, People’s Republic of China

## Abstract

This retrospective and a single-center study evaluated the prognostic value of the maximum serum creatinine value (maxCr) and the maximum serum creatinine growth rate (Vmax) after paraquat (PQ) ingestion. One hundred and seventy-one patients with PQ poisoning were treated with a uniform protocol. Demographic variables, clinical manifestations, relevant laboratory data, maxCr and Vmax of all patients were recorded and calculated. The time after PQ ingestion of maxCr and Vmax were also recorded. Vmax and MaxCr exhibited statistically significant differences between the survivor (*n* = 53) and death (*n* = 118) groups. Vmax appeared earlier in the death group than the survivor group. Regard to the receiver operating characteristic (ROC) curve analysis, Vmax had an AUC of 0.861 (*95% CI*, 0.801–0.921) and the optimal cut-off value of 6.21 μmol/(L·h) (sensitivity, 76.3%; specificity, 81.1%). MaxCr had an area under the curve (AUC) of 0.821 (95*% CI*, 0.752–0.889) and the optimal cut-off value of 225.5 µmol/L (sensitivity, 82.2%; specificity, 67.9%). The comparison of the AUC in the two parameters showed no significant difference, but Vmax appeared earlier than maxCr. Based on binary logistic regression analysis, MaxCr and Vmax both showed strong predictive powers for evaluating the prognosis of acute PQ poisoning patients.

## Introduction

Paraquat (PQ) is a non-selective contact herbicide which has been world-widely used for many years, especially in the developing agricultural countries, including China. Due to its high efficiency and low residue in crops, it becomes a good choice for weed killing^1^. PQ has the characteristics of high toxicity, easy to obtain and no specific antidote. Many people succumb to PQ poisoning each year deliberately or unintentionally^2,3^. Upon ingestion, PQ is mostly distributed in the lungs^1^. Ingestion of approximately 40 mL of a 24% PQ solution may cause the patients to die of multiple organ failure in the next few hours or days, including lungs, liver, heart and kidney failure. Smaller exposure of PQ is likely to result in the death from progressive pulmonary fibrosis and respiratory failure in the following weeks^4,5^. Although many treatments have been applied to the patients, including adsorbent, hemoperfusion, antioxidant, immunosuppressive therapy and respiratory support in clinical practice, the mortality still remains very high^6^. Therefore, reliable and effective predictors of prognosis may be necessary to guide the treatments and future clinical research on specific antidotes and other therapies^7^. To date, several prognostic predictors have been reported to predict the outcome of acute PQ poisoning, such as patients’ plasma or urine PQ concentration, arterial lactate, abnormal pancreatic enzymes, hematological parameters, PQ ingestion volume, acute physiology and chronic health evaluation (APACHE) II score, sequential organ failure assessment (SOFA) score^8–19^. However, these indicators above have deficiencies to accurately predict prognosis, because of the limited availability and reliability. Thus, a better alternative prognostic indicator is still needed for the patients with acute PQ poisoning in clinical practice.

The serum creatinine concentration (sCr) is a sensitive marker to reflect the acute kidney injury (AKI)^20^. It has also been demonstrated as a good prognostic marker in many diseases including acute coronary syndromes, sepsis, cirrhotic, etc^21–23^. PQ is excreted in the urine within 12–24 hours after ingestion, without undergoing further metabolism. In addition, PQ can lead to renal injury in the early stage of poisoning^24,25^. In this study, we explored the prognostic values of the maximum serum creatinine value (maxCr) and the maximum serum creatinine growth rate (Vmax) after PQ poisoning, in order to evaluate whether these two indicators could be alternative predictive markers.

## Results

### Baseline characteristics

After screening, 171 eligible patients were selected in this study, which contained 83 males and 88 females, 53 (30.99%) patients survived during the study while 118 (69.01%) patients who died of PQ poisoning. The patients reached our hospital at a median time of 6.00 hours (quartile range 4.00 to 24.00) in the survivor group and 5.00 hours (quartile range 3.88 to 11.25) in the death group, with no statistical significance. The median sCr at admission was 65.00 μmol/L (quartile range 48.00 to 100.00) and 72.00 μmol/L (quartile range 55.75 to 105.25) in the survivor and death groups, respectively (*P* = 0.127). Most of the patients vomited after PQ ingestion. In the survivor group, 30 (56.60%) patients had inflammation of the oral mucosa and 12 (22.60%) patients had epigastric pain. In the death group, 62 (52.50%) patients had inflammation of the oral mucosa and 41 (34.70%) patients had epigastric pain. In brief, clinical manifestations had no statistical differences between these two groups (all *P* > 0.05). No differences in sex ratio, age, rates of smokers, drinkers, time to hospital arrival, heart rate, respiratory rate, partial pressure of oxygen (PaO_2_) and serum potassium were found between the survivor and death groups (all *P* > 0.05). Comparing with survivors, the deaths had significantly lower arterial PH and partial pressure of carbon dioxide (PaCO_2_), but higher ingestion amount, APACHE II score, basal body temperature, mean arterial blood pressure (MAP), white blood cells count and serum sodium. Patients in the death group represented a higher Vmax of (9.70 ± 4.60) μmol/(L·h) and a higher maxCr of (372.16 ± 149.28) μmol/L when compared to the survivor group. Moreover, Vmax appeared at a median time of 41.15 hours (quartile range 30.97 to 59.41) in the survivor group and 30.03 hours (quartile range 21.09 to 43.49) in the death group, which showed that Vmax of the death group reached peak earlier than the survivor group (Table 1). And the time to Vmax [34.32 hours (quartile range 23.25 to 48.30)] was shorter than the time of maxCr [58.63 hours (quartile range 38.38 to 85.37)] for all PQ poisoning patients (*P* < 0.001).

**Table 1:** Comparisons of baseline characteristics between the survivor and death groups with acute PQ poisoning.

Parameters	Survivor group (*n* = 53)	Death group (*n* = 118)	*P* value
Sex (male)	20 (37.70%)	63 (53.40%)	0.069
Age (y)	31.00 (23.00, 43.50)	33.50 (24.00, 49.00)	0.375
Time to hospital arrival (h)	6.00 (4.00, 24.00)	5.00 (3.88, 11.25)	0.325
Smoking status			0.883
Never smoker	39 (73.6%)	86 (72.9%)	
Current smoker	12 (22.6%)	29 (24.6%)	
Former smoker	2 (3.8%)	3 (2.5%)	
Drinking status			0.476
Never drinker	44 (83.0%)	93 (78.8%)	
Current drinker	9 (17.0%)	22 (18.6%)	
Former drinker	0 (0.00%)	3 (2.5%)	
Estimated ingestion amount (mL)	10.00 (5.00, 25.00)	30.00 (20.00, 100.00)	<0.001
Clinical manifestation			
Vomiting	47 (88.70%)	108 (91.50%)	0.577
Inflammation of the oral mucosa	30 (56.60%)	62 (52.50%)	0.740
Epigastric pain	12 (22.60%)	41 (34.70%)	0.152
Admission sCr (μmol/L)	65.00 (48.00, 100.00)	72.00 (55.75, 105.25)	0.127
Vmax [μmol/(L·h)]	4.04 ± 3.31	9.70 ± 4.60	<0.001
MaxCr (μmol/L)	206.11 ± 116.67	372.16 ± 149.28	<0.001
Time to Admission sCr (h)	6.17 (4.28, 24.35)	5.99 (4.15, 12.26)	0.985
Time to Vmax (h)	41.15 (30.97, 59.41)	30.03 (21.09, 43.49)	<0.001
Time to maxCr (h)	62.87 (43.07, 81.09)	57.21 (36.65, 88.72)	0.281
Body temperature (°C)	37.11 ± 0.44	37.31 ± 0.74	0.031
Heart rate (beats/min)	82.55 ± 14.05	85.54 ± 17.03	0.264
Respiratory rate (breaths/min)	20.00 (18.00, 20.00)	20.00 (18.75, 21.00)	0.268
Mean arterial pressure (mm Hg)	87.48 ± 14.05	92.57 ± 13.51	0.026
PH	7.44 (7.41, 7.46)	7.41 (7.37, 7.44)	<0.001
PaO_2_ (mm Hg)	98.25 ± 19.10	98.57 ± 21.10	0.924
PaCO_2_ (mm Hg)	33.86 ± 5.19	30.07 ± 7.97	<0.001
Potassium (mol/L)	3.53 ± 0.45	3.44 ± 0.57	0.325
Sodium (mol/L)	139.00 (137.00, 141.00)	140.00 (138.00, 143.00)	0.001
White blood cells (×10^9^/L)	12.10 (9.85, 14.10)	17.25 (14.23, 23.23)	<0.001
APACHE II score	4.85 ± 3.44	8.73 ± 4.73	<0.001

PQ: paraquat; sCr: the serum creatinine concentration; Vmax: the maximum serum creatinine growth rate; maxCr: the maximum serum creatinine value; PaCO_2_: partial pressure of carbon dioxide; PaO_2_: partial pressure of oxygen; APACHE II: the acute physiology and chronic health evaluation II.

### Binary logistic regression analysis

According to the initial analysis outcomes, Vmax, maxCr and APACHE II score, with statistical significance, were selected to perform binary logistic regression analysis for death prediction in patients with PQ poisoning. As shown in Table 2, we could consider that the increase of these parameters had been associated with a significantly higher risk of mortality in PQ poisoning population. The following equation was derived: Logit (p) = 0.120 × APACHE II score + 0.005 × maxCr + 0.251 × Vmax − 2.926, where the probability of survivors = 1/[1 + e^Log it (p)^].

**Table 2:** Binary logistic regression analysis of maxCr, Vmax and APACHE II after acute PQ poisoning.

Parameters	B	Standard error	Wals *χ*^2^ value	*P* value	Odds ratio
maxCr	0.005	0.002	4.725	0.030	1.005
Vmax	0.251	0.084	8.819	0.003	1.285
APACHE II	0.120	0.056	4.647	0.031	1.128
Constant	−2.926	0.603	23.548	<0.001	0.054

PQ: paraquat; Vmax: the maximum serum creatinine growth rate; maxCr: the maximum serum creatinine value; B: regression coefficient; APACHE II: the acute physiology and chronic health evaluation II.

### ROC curve analysis

**Figure 1:**
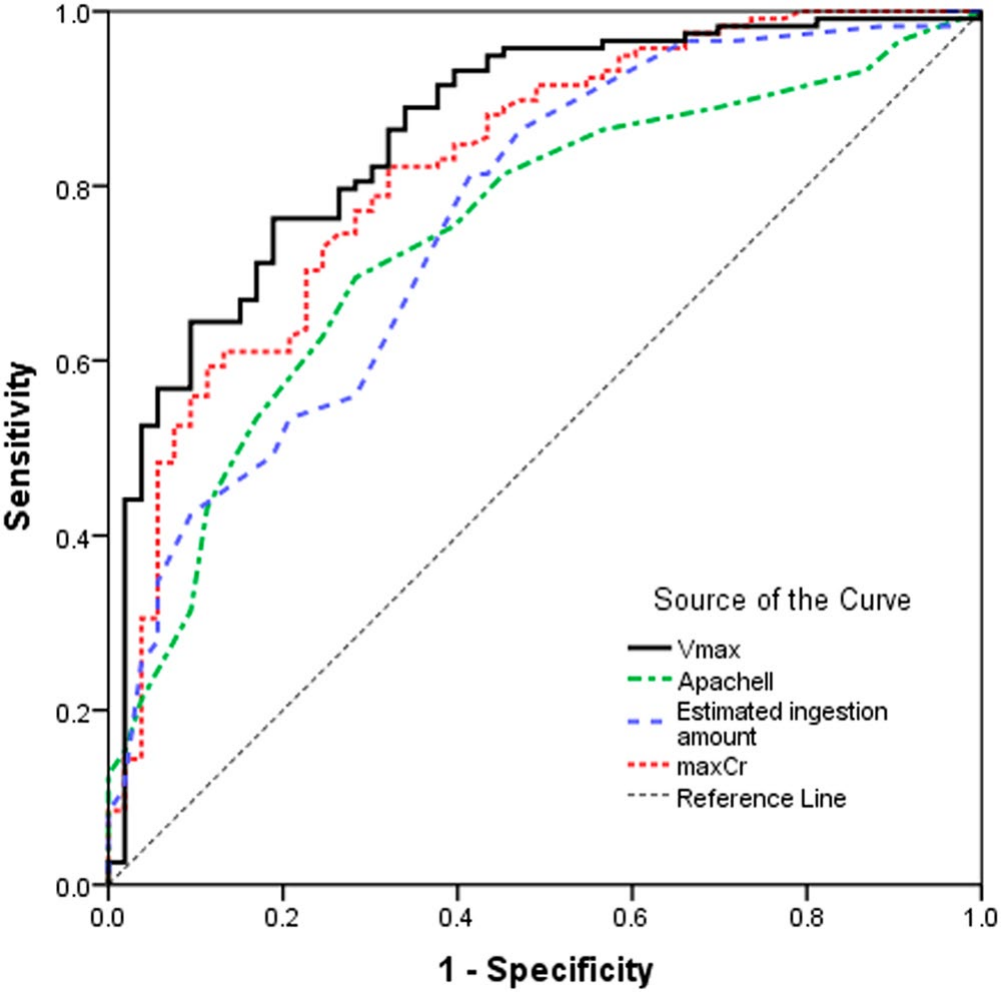
Receiver operating characteristic curves of maxCr, Vmax, APACHE II and estimated Ingestion amount. Vmax: the maximum serum creatinine growth rate; maxCr: the maximum serum creatinine value; APACHE II: the acute physiology and chronic health evaluation II score.

To further evaluate the predictive value of these two parameters, ROC curves were drawn and the areas under the curve (AUC) were calculated for maxCr and Vmax. Table 3 and Fig. 1 demonstrated that maxCr had an AUC of 0.821 (95*% CI*, 0.752–0.889) and the optimal cut-off value of 225.5 μmol/L (sensitivity, 82.2%; specificity, 67.9%); while Vmax had an AUC of 0.861 (95*% CI*, 0.801–0.921) and the optimal cut-off value of 6.21 μmol/(L·h) (sensitivity, 76.3%; specificity, 81.1%). That means both maxCr and Vmax could be applied to predict clinical outcome (survivor or death) in the patients with PQ poisoning. Since maxCr appeared relatively late, it might be not appropriate to predict early prognosis, but could has referential value in the late stage of PQ poisoning.

**Table 3:** Receiver operating characteristic curve analysis of maxCr, Vmax, APACHE II score and estimated Ingestion amount after acute PQ poisoning.

Parameters	AUC	Cut-off Value	95*% CI*	Sensitivity	Specificity
Vmax	0.861	6.21	0.801 to 0.921	0.763	0.811
maxCr	0.821	225.50	0.752 to 0.889	0.822	0.679
APACHE II	0.745	6.50	0.667 to 0.822	0.695	0.717
Estimated ingestion amount of PQ	0.762	18.75	0.684 to 0.839	0.814	0.585

PQ: paraquat; Vmax: the maximum serum creatinine growth rate; maxCr: the maximum serum creatinine value; AUC: area under the curve; CI: confidence interval; APACHE II: the acute physiology and chronic health evaluation II.

In order to compare Vmax and maxCr with the other traditional prognostic indicators, we explored the predictive efficiency of APACHE II score and PQ estimated ingestion amount basing on the same patients. As shown in Fig. 1 and Table 3, the APACHE II score had an AUC of 0.745 (95*% CI*, 0.667–0.822) and the optimal cut-off value was 6.5 (sensitivity, 69.5%; specificity, 71.7%). The AUC of PQ ingestion amount was 0.762 (95*% CI*, 0.684–0.839) and the the optimal cut-off value was 18.75 mL (sensitivity, 81.4%; specificity, 58.5%). For further determine which one had a higher value of predictive power, the pair comparison of the AUC for these parameters was performed. As shown in Table 4, Vmax had a higher AUC with statistical significance, compared with APACHE II score and PQ ingestion amount, but had no statistically significant difference (*P* = 0.108) compared with maxCr. Table 4 also revealed no statistically significant difference among maxCr, APACHE II score and PQ ingestion amount. These results demonstrated that Vmax could be a better prognostic indicator in acute PQ poisoning.

**Table 4:** Pair comparison of the AUC among maxCr, Vmax, APACHE II and estimated Ingestion amount in acute paraquat poisoning patients.

Pair	Area Difference	95*% CI*	*P* value
Vmax, maxCr	0.040	−0.009 to 0.089	0.108
Vmax, APACHE II	0.116	0.037 to 0.196	0.004
Vmax, Estimated Ingestion amount	0.099	0.019 to 0.180	0.016
maxCr, APACHE II	0.076	−0.009 to 0.161	0.078
maxCr, Estimated Ingestion amount	0.059	−0.033 to 0.152	0.208
APACHE II, Estimated Ingestion amount	−0.017	−0.108 to 0.074	0.716

Vmax: the maximum serum creatinine growth rate; maxCr: the maximum serum creatinine value; CI: confidence interval; APACHE II: the acute physiology and chronic health evaluation II.

### Survival analysis

**Figure 2:**
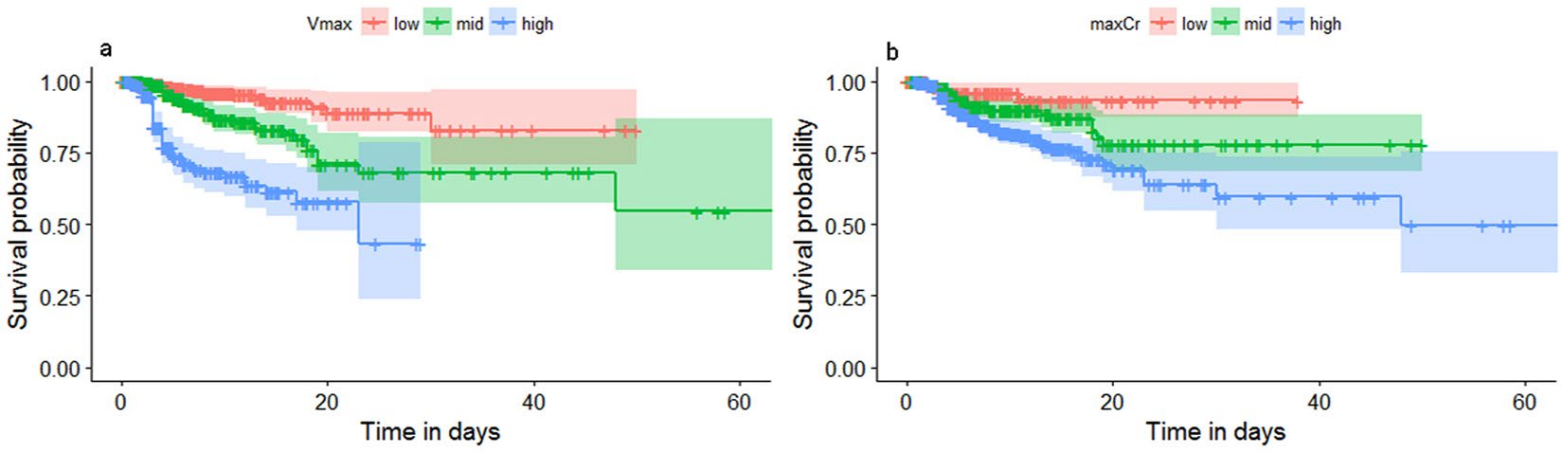
Kaplan-Meier survival curves of Vmax (**a**) and maxCr (**b**) in patients with paraquat poisoning. Vmax: the maximum serum creatinine growth rate; maxCr: the maximum serum creatinine value.

To further explore the correlation between Vmax, maxCr and mortality, Kaplan-Meier survival curves are depicted in Fig. 2. In Fig. 2a, the patients were divided into the low (0–5 μmol/L), middle (5–10 μmol/L) and high (>10 μmol/L) groups according to Vmax values. In Fig. 2b, the patients were divided into the low (0–150 μmol/L), middle (150–300 μmol/L) and high (>300 μmol/L) groups according to maxCr values. As shown in Fig. 2, the higher Vmax and maxCr groups had lower survival probabilities, demonstrating that both Vmax and maxCr were related to the mortality of patients after PQ poisoning.

## Discussion

Acute PQ poisoning is still a major public health problem in many developing countries with a high mortality. Generally, the prognosis of PQ poisoning patients mainly depends on ingestion volume. However, accurate PQ ingestion volume is difficult to estimate and many patients vomited after ingestion. Several studies demonstrated that serum PQ concentration was a reliable marker for predicting death in the patients with PQ poisoning^9–11^. But not all hospitals may be able to detect plasma PQ concentration right away. It is reported that PQ is selectively distributed in the lungs and slowly eliminated, which makes its concentration in the lungs more than 10 times higher than that in the plasma^26^. PQ causes acute pulmonary edema, hemorrhage, interstitial inflammation and alveolar epithelium damage; ultimately, it developed progressively to severe lung fibrosis and respiratory failure within a few weeks. This is the most common cause of death after PQ poisoning^27–30^. For this reason, the degree of the lung injury may be beneficial to predict prognosis. However, several limitations make it difficult to be a prognostic indicator, including the various pathological characteristics of lungs, the lack of effective hematological indexes and the inaccessibility of the sufficient imageological diagnosis in acute PQ poisoning patients. Thus, we have to search for other potential indexes which are valuable, simple and earlier accessible.

According to PQ toxicokinetics, it is excreted in the urine without further metabolism in the first 12–24 hours^24^. Impaired kidney function may reduce the excretion of poison, increase its accumulation and toxic potential in the other organs^31^. Although the mechanism whereby PQ leading to AKI has not yet been fully understood, previous studies illustrated that PQ could accumulate inside renal tubular cells, generate large reactive oxygen species (ROS), deplete nicotinamide adenine dinucleotide phosphate hydrogen (NADPH), damage mitochondrion, as well as arising lipid peroxidation and oxidation of proteins, carbohydrates, DNA and sulphide groups, ultimately result in the injury of proximal tubules^1,31^. Mohamed *et al*.^32^ manifested that markers for kidney injury such as neutrophil gelatinase-associated lipocalin (NGAL) could be up-regulated in AKI. However, these indicators could not predict death independently. Only functional markers such as sCr possessed much better predictive power than others for mortality after PQ poisoning. Moreover, reduced kidney function is the only factor that results in a rapid rise of serum creatinine after PQ poisoning^31^. Actually, many studies had demonstrated that sCr increased early after PQ poisoning and the predictive value of sCr had been explored previously^24,25,32–35^. These evidences supported our hypothesis that maxCr and Vmax might be the optimal predictive indicators for death in patients with PQ poisoning.

A previous report by Ragoucy-Sengler and Pileire^35^ had a hypothesis that the rate of change in serum creatinine concentration might be beneficial to predict death in PQ poisoning patients. Roberts *et al*. confirmed this hypothesis by a small prospective study containing 20 patients with PQ poisoning. They demonstrated that the rate of increase in creatinine over the first 24 hours might possess the prognostic utility for outcomes after PQ poisoning^33^. In the present study, 171 recorded patients met the inclusion criteria and were performed analysis. We used binary logistic regression analysis and identified three independent factors including APACHE II, Vmax and maxCr for prediction of PQ poisoning patients, the following equation was derived: Logit (p) = 0.120 × APACHE II score + 0.005 × maxCr + 0.251 × Vmax − 2.926, where the probability of survivors = 1/[1 + e^Log it (p)^]. The present study also found that APACHE II score and ingestion amount of PQ both had significant differences between the survivors and deaths. And ROC curve analysis revealed their prognostic values after PQ poisoning, which were in accordance with some previous studies that APACHE II score and ingestion amount of PQ had good prognostic values for acute PQ poisoning^15–18^. Moreover, Vmax may have a better predictive power than APACHE II score and ingestion amount of PQ by comparison of the AUC values. Vmax had no statistical difference of AUC compared with maxCr. However, Vmax appeared earlier than maxCr after PQ poisoning. Therefore, both Vmax and maxCr were correlated with the mortality. Vmax had an effective prognostic value after acute PQ poisoning in the early stage. While the predictive value of maxCr was embodied in the later stage of PQ poisoning, with a limited utility in the early stage. Perhaps it could be used for these patients who can not arrived the hospital in the early stage of PQ poisoning.

The present study reported a relatively novel method to predict prognosis of PQ poisoning patients by maxCr and Vmax. On the basis of raw data, we got a new variable, Vmax, which could predict clinical outcome earlier than maxCr in the early stage of acute PQ poisoning. The binary logistic regression analysis, survival curve analyses, combined with ROC analyses provided enough evidences that maxCr and Vmax both had the independently strong prognosis predictive power after PQ ingestion. Moreover, the measurement of sCr is simple, routine and inexpensive, so almost every hospital can easily acquire the data of maxCr and Vmax.

Inevitably, there are some limitations in our study. The major limitation was the absence of a quantitative plasma or urine PQ concentration due to the deficiency of testing devices in our hospital during the period of this study. And the present study was a retrospective study and only 171 patients met inclusion criteria in a single institution. These results need to be further validated in a large population of multi-center data.

In conclusion, both the maxCr and Vmax have significantly effective values to predict prognosis for death after PQ poisoning. It is likely that Vmax can predict death in the acute PQ poisoning, earlier than maxCr. MaxCr has a predictive power in the later stage of PQ poisoning. If these results are confirmed properly, the two parameters would be applied to clinical practice for PQ poisoning patients, predicting death, guiding treatments and supporting the search of new efficient therapies.

## Materials and Methods

### Study population

This retrospective cohort study enrolled 255 patients with PQ poisoning who were admitted to the First Affiliated Hospital of Zhejiang University from May 2011 to July 2017. The semi-quantitative urine dithionite PQ tests had been applied to all these patients to make a definite diagnosis of PQ poisoning along with medical history, physical exam and other laboratory exams at admission. The first blood sample was collected on admission, and blood samples were collected in the morning routinely during hospitalization to detect sCr and other laboratory indexes. All enrolled patients had ingested PQ and had no history of severe diseases of heart, lungs, liver and kidneys. The patients who met any of the following criteria were excluded: taking other medicines or poisons (*n* = 4); sCr was in normal range during hospitalization (*n* = 66); sCr had reached peak when admission (*n* = 14). Notably, 8 patients were died of the 14 patients. Finally, a total of 171 patients were eligible in this study, which were classified into the two groups (the survivor group and death group), depending on the condition of patient at hospital discharge and follow-up after 3 months.

### Data collection

The data of all patients were recorded, including the following: (1) Demographic variables such as age, sex, smoking status and drinking status, (2) admission time, time to hospital arrival, estimated ingestion amount of 20% PQ solution, (3) clinical manifestations, (4) sCr during hospitalization, time to sCr test, (5) basal body temperature, heart rate, respiratory rate, MAP, arterial pH, PaO_2_, PaCO_2_, white blood cells count, serum sodium, serum potassium and APACHE II score within the 24 h after admission. The maxCr and Vmax were recorded and calculated respectively. The time after PQ ingestion of maxCr and Vmax were also recorded. Notably, Vmax was calculated by the following formula: Vmax = Max [(C2 − C1)/(T2 − T1)]. C1 and C2 were the adjacent serum creatinine values, T1 and T2 were the serum creatinine testing time after PQ poisoning corresponding to C1 and C2. After calculation, the maximum value was taken as Vmax, the corresponding T2 was defined as the time of Vmax.

### Treatments

All the patients were treated with a uniform protocol at the time of hospitalization, which included the prevention of PQ continuing absorption, intensive hemoperfusion, antioxidant, immunosuppressive therapy and respiratory support by mechanical ventilation or extracorporeal membrane oxygenation (ECMO).

### Statistical analysis

SPSS statistical software package (version 21.0, SPSS Inc., IBM, Chicago, IL, USA), Sigma plot software (version 12.0, Systat Software Inc., San Jose, CA, USA) and R software (version 3.3.0, R Foundation for Statistical computing, Vienna, Austria) were used to perform data analysis. Data were expressed as mean ± standard deviation for normal distribution variables or median with the quartile range for skewed distribution variables. The independent two-sample *t*-test or a Mann-Whitney U-test or Chi-square test was used to analyse the statistically significant differences between the survivor and death groups. Additionally, the binary logistic regression analysis was used to select and determine the predictive factors. Receiver operating characteristic (ROC) curve was performed to determine the predictive powers of maxCr, Vmax, estimated PQ ingestion amount and APACHE II score. The predictive powers of the parameters were also compared by Sigma plot 12.0 software. Kaplan-Meier survival curves were depicted for Vmax and maxCr. All *P* values were two tailed and *P* < 0.05 was considered as significant.

### Ethics statement

This study was approved by the Ethical Committee of the First Affiliated Hospital, School of Medicine, Zhejiang University. We obtained written informed content for all enrolled patients. The treatment protocols were carried out in accordance with the principles of the Helsinki Declaration.
